# Effect of free fatty acids on TGF-β1 mediated fibrogenesis in hepatic stellate cells

**DOI:** 10.1016/j.molmet.2025.102309

**Published:** 2025-12-17

**Authors:** William De Nardo, Jacqueline Bayliss, Sheik Nadeem Elahee Doomun, Olivia Lee, Paula M. Miotto, Natasha D. Suriani, Shuai Nie, Michael Leeming, Diego A. Miranda, David P. De Souza, Matthew J. Watt

**Affiliations:** 1 Department of Anatomy and Physiology, School of Biomedical Sciences, Faculty of Medicine, Dentistry & Health Sciences, The University of Melbourne, Melbourne, Victoria, 3010, Australia; 2 Drug Delivery, Disposition and Dynamics, Monash Institute of Pharmaceutical Sciences, Parkville, Victoria, Australia; 3 Metabolomics Australia, Bio21 Institute, University of Melbourne, Melbourne, Australia; 4 Melbourne Mass Spectrometry and Proteomics Facility, The Bio21 Molecular Science and Biotechnology Institute, The University of Melbourne, Melbourne, Victoria, 3010, Australia; 5 Gilead Sciences, Foster City, CA, 94404, USA

**Keywords:** Hepatic stellate cell, Lipid metabolism, Liver fibrosis, Collagen, Metabolic reprogramming, MASLD, Palmitate

## Abstract

**Abstract/objective:**

Metabolic associated steatotic liver disease (MASLD) is the most prevalent liver disorder and a major risk factor for hepatic fibrosis. Activated hepatic stellate cells (HSCs) are the primary source of collagen production in the liver, contributing to fibrosis. However, the mechanisms by which HSCs reprogram their metabolism to support sustained collagen production, particularly in a lipid-rich environment such as MASLD, remain inadequately understood. In this study, we investigated the effect of extracellular fatty acids on HSC substrate metabolism, HSC activation, and collagen synthesis.

**Methods:**

Immortalized human HSCs (LX-2 cells) were cultured with or without transforming growth factor-beta 1 (TGF-β1) and varying concentrations of palmitate or oleate. Cellular lipid composition was assessed by mass spectrometry lipidomics. Fatty acid metabolism was assessed using radiometric techniques and isotopic labelling experiments using ^13^C-glucose or ^13^C-palmitate. HSC activation was assessed by measuring *ACTA2, TGFB1, and COL1A1* mRNA levels and collagen secretion by ELISA.

**Results:**

TGF-β1 reduced the abundance of many lipid types in LX-2 cells. Exogenous palmitate did not increase HSC activation, as determined by *ACTA2, TGFB1, COL1A1* mRNA levels. Palmitate potentiated TGF-β1 induced collagen secretion but not in the presence of oleate. Palmitate reduced glucose incorporation into glycine in activated HSCs and induced a reciprocal increase in palmitate incorporation into glycine, most likely via carbons derived from TCA cycle intermediates. Pharmacological inhibition of fatty acid uptake reduced TGF-β1-mediated collagen secretion.

**Conclusions:**

These results suggest that in activated HSCs, palmitate oxidation is reduced and that TCA cycle intermediates derived from palmitate are used as carbon sources for amino acid production that supports collagen synthesis and secretion.

## Highlights

• TGF-β1 remodels fatty acid metabolism and the lipidome of activated hepatic stellate cells (HSCs).
• Fatty acids do not increase TGF-β1 mediated HSC activation.
• Carbons derived from palmitate are used to synthesise amino acid precursors in activated HSCs.
• Palmitate reduces the incorporation of glucose derived carbons into amino acids.
• Inhibiting fatty acid uptake reduces collagen secretion.

## Graphical abstract

**Figure:**
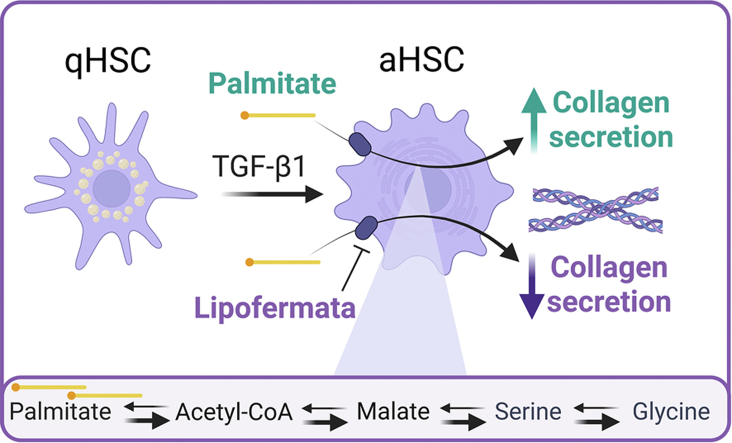

## 1 Introduction

Metabolic dysfunction associated steatotic liver disease (MASLD) is the most common liver disease with an estimated global prevalence of 32.4% [1]. Metabolic dysfunction associated steatotic liver (MASL) is defined by excessive storage of lipid within hepatocytes, with or without lobular inflammation, and a subset of patients will progress to metabolic-associated steatohepatitis (MASH), that is further characterised by hepatocellular ballooning [2]. Liver fibrosis is present in 42–64% of individuals with MASLD [3] and regardless of the severity of MASLD, fibrosis significantly increases the risk of progression to cirrhosis and hepatocellular carcinoma [3,4], and is the primary predictor of mortality in individuals with MASLD [[5], [6], [7]].

Hepatic fibrogenesis is a complex process involving interactions between a variety of resident and non-resident liver cell populations that leads to excessive accumulation of extracellular matrix (ECM), causing a loss of tissue integrity and impaired liver function. Hepatic stellate cells (HSCs) are the primary mediators of hepatic fibrogenesis. HSCs are typically quiescent and maintain ECM homeostasis and sinusoidal blood flow. However, in MASLD, a variety of inflammatory, metabolic and damage-associated signals cause HSC activation, loss of retinol-containing lipid droplets (LD), and transdifferentiation into a myofibroblast-like phenotype. Although various factors contribute to the differentiation of HSCs into myofibroblasts, transforming growth factor-β1 (TGF-β1) is the primary driver of fibrogenesis [[8], [9], [10]]. Activated HSCs secrete ECM proteins, including collagen type one α1 chain (COL1A1), which is the major ECM protein in liver fibrosis [4,11].

HSCs reprogram their metabolism to meet the increased demand for collagen synthesis and to accommodate cellular processes associated with their activation, including proliferation, contractility and migration [12,13]. Glutaminolysis is the process where glutamine is converted into glutamate, which can be further converted into α-ketoglutarate (αKG) to power the tricarboxylic acid (TCA) cycle for ATP synthesis [12,13]. Glutaminolysis is increased with HSC activation [14] and pharmacological inhibition of glutaminase-1, the rate-limiting enzyme for this process, reduces HSC activation [14]. Similarly, pharmacological inhibition of glutamate dehydrogenase prevents the conversion of glutamate to αKG and reduces HSC activation [15], highlighting the importance of glutamine metabolism for HSC-mediated fibrosis.

Activated HSCs also upregulate glucose transporters and rate-limiting enzymes of glycolysis, including hexokinase-2 and fructose-2,6-bisphosphatase-3 (PFKFB3), to increase ATP synthesis [16]. Inhibition of glycolysis by 2-deoxyglucose or galloflavin reduces HSC activation [16]. Aside from providing substrate to fuel ATP synthesis, glycolysis provides intermediates for biosynthetic pathways, including *de novo* serine and glycine synthesis that is required for collagen production [17]. Indeed, diversion of carbons from glucose to glycine contained within COL1A1 protein is increased in TGF-β1–stimulated lung fibroblasts [18] and blocking the initial step of serine synthesis by phosphoglycerate dehydrogenase inhibition reduces collagen production [17]. This process may be conserved in HSCs. Moreover, *de novo* lipogenesis (DNL) appears to play a crucial role in HSC activation. DNL is the process where carbohydrate or amino acid-derived citrate from the TCA cycle is converted into acetyl-CoA, which is then transformed into malonyl-CoA by acetyl-CoA carboxylase (ACC) and subsequently into palmitate by fatty acid synthase (FASN) [19]. The genes that encode DNL enzymes are increased with HSC activation [20,21] and ACC inhibition directly impairs the profibrogenic activity of HSCs [22], although the underlying mechanisms remain unresolved.

A hallmark of HSC activation is the rapid loss of retinol containing LD [23], however, less is known about other aspects of fatty acid metabolism in HSC activation. It is well documented that fatty acid supply to the liver is increased in MASLD [[24], [25], [26]], positioning fatty acids as an abundant carbon source for hepatocytes and other liver cell types including HSCs. Fatty acids were shown to be important for energy production as activated HSCs upregulate carnitine palmitoyl transferase 1 (CPT1), the enzyme responsible for mitochondrial fatty acid transport, and *Cpt1* deletion in HSCs reduces HSC activation and fibrogenesis in mice [27]. However, extensive characterization of fatty acid metabolism in MASLD has largely been focused on whole liver, hepatocytes, liver homogenates, or precision-cut liver slices [[28], [29], [30]], necessitating a deeper understanding of fatty acid metabolism in activated HSCs.

The purpose of this study was to determine the fate of extracellular-derived non-esterified fatty acids on HSC activation and fibrogenesis. We combined radiolabelled isotope metabolic tracing, stable isotope metabolomics analysis and mass spectrometry lipidomics in HSCs to demonstrate that lipid metabolism is altered with HSC activation and that HSCs utilise palmitate to support collagen production and fibrogenesis.

## 2 Material and methods

### 2.1 Patient recruitment and collection of liver secreted factors

Participants provided written and verbal informed consent. The study conformed to the ethical guidelines of the 1975 Declaration of Helsinki, was approved by the University of Melbourne Human Ethics Committee (ethics ID 1851533), The Avenue Hospital Human Research Ethics Committee (Ramsay Health; ethics ID WD00006, HREC reference number 249), the Alfred Hospital Human Research Ethics Committee (ethics ID GO00005), and Cabrini Hospital Human Research Ethics Committee (ethics ID 09-31-08-15).

The detailed procedure describing patient recruitment, blood collection, and precision-cut liver slicing were reported previously [31]. Briefly, individuals undergoing bariatric surgery for obesity were fasted overnight, venous blood was collected and analysed by Melbourne Pathology. A liver wedge biopsy was procured from patients, precision cut to 250 μm using a Krumdieck tissue slicer (TSE Systems), and incubated in EX-CELL 325 protein free medium (Sigma–Aldrich, Australia) for 16 h at 37 °C. The incubation medium was collected, centrifuged at 300 x g for 10 min at 4 °C and the supernatant was snap-frozen at −80 °C.

### 2.2 Cell culture and maintenance

LX-2 human hepatic stellate cells (#SCC064, Merck) were maintained in high glucose DMEM, supplemented with 2% FBS, 1% penicillin-streptomycin and 1X Glutamine (Millipore Cat. No. TMS-002-C) unless specified otherwise. All cells were maintained in a humidified incubator at 37 °C and 5% CO_2_. Cells were seeded in high glucose DMEM, supplemented with 10% FBS for 24 h, then serum starved in high glucose DMEM for 24 h to induce quiescence [[32], [33], [34]], and then incubated with high glucose DMEM with or without 5 ng/mL of TGF-β1 (R&D systems) for a specified period of time.

### 2.3 Metabolic tracing assays

#### 2.3.1 Fatty acid metabolism using ^14^C tracers

LX-2 cells were plated and assessed for fatty acid metabolism as previously described [30], with few exceptions. Following serum starvation, cells were incubated with or without 5 ng/mL of TGF-β1 (R&D systems) for 16 h, followed by a 2-hour incubation in cell culture medium containing 500 μM palmitate and 1 μCi/mL [1-^14^C] palmitate (NEC075H250UC; PerkinElmer) or 500 μM oleate and 1 μCi/mL [1-^14^C] oleate (NEC317250UC; PerkinElmer) conjugated to 2% bovine serum albumin and 1 μM l-carnitine. Following 2 h incubation, fatty acid oxidation was assessed as the sum of ^14^CO_2_ production in the culture medium and ^14^C-palmitate degradation into acid soluble metabolites in LX-2 cells. The protein concentration was assessed using Pierce™ BCA protein assay kit (Thermo Scientific, #23225, USA). Lipids were extracted using (2:1; v:v) chloroform:methanol mixture, separated using thin layer chromatography (TLC) and incorporation of ^14^C-palmitate into lipid classes was determined by radioactivity assessed using TriCarb® 2810 TR liquid scintillation analyser (PerkinElmer, USA) as previously described [30]. Total fatty acid uptake was assessed as the sum of fatty acid oxidation and storage into lipid pools.

#### 2.3.2 Isotopic tracing assays and semi-targeted metabolomics

To enable sufficient enrichment of labelled palmitate-derived carbons within intracellular amino acid pools, LX-2 cells were seeded in 6 well plates overnight, serum starved for 24 h in Minimum Essential Medium (MEM) (Gibco, Cat: 41090), treated with or without 5 ng/mL of TGF-β1 for 24 h, and then incubated with MEM supplemented with 1 μM l-carnitine, 2% BSA, 0.5 mM [U-^13^C]palmitate (Cambridge Isotope Laboratories, catalog no.: CLM-409) or 20 mM [U-^13^C]glucose (Sigma, catalog no.: 389374) contained in the MEM (total glucose concentration 25 mM) with or without 5 ng/mL of TGF-β1.

For studies using human liver secreted medium, isotopic tracing of LX-2 cells was performed as described above with few exceptions. Following 24 h serum starvation in MEM, cells were incubated in 50% MEM and 50% liver-secreted medium from individuals with or without histologically confirmed MASLD, that was supplemented with 2% BSA, 1 μM l-carnitine, 0.5 mM [U-^13^C] palmitate (Cambridge Isotope Laboratories, catalog no.: CLM-409) for 24 h.

A monophasic extraction protocol was used to extract the metabolites from the LX-2 cells. To the adherent cells, 600 μL of chilled 9:1 methanol/chloroform containing 0.5 nmol of ^13^C_6_ scyllo-inositol was added. Each sample was scraped for 30 s to rupture and release the cells off the plate. The content of 2 wells were combined to create one replicate (total 12 wells per condition). The cell lysate was transferred into 1.5 mL Eppendorf tube, vortexed and then incubated at 4 °C for 10 min with continuous agitation (12 *g*) using an Eppendorf Thermomixer C. The samples were centrifuged at 4 °C for 10 min at 16,000×*g* using an Eppendorf centrifuge 5430 R. The supernatant was transferred into a fresh 1.5 mL Eppendorf tube and the cell debris was discarded. Four hundred and fifty μL of each study sample was transferred into HPLC inserts and evaporated at 30 °C to complete dryness, using a CHRIST RVC 2–33 CD plus speed vacuum. To limit the amount of moisture, present in the insert, 50 μL 100% methanol (LCMS grade) was added to each insert and evaporated using a speed vacuum.

##### 2.3.2.1 Metabolomics: sample derivatisation

Dried samples for the GCMS analysis were derivatised online using the Shimadzu AOC6000 autosampler robot. Derivatisation was achieved by the addition of 25 μL methoxyamine hydrochloride (30 mg/mL in pyridine, Merck) followed by shaking at 37 °C for 2 h. Samples were then derivatised with 25 μL of N,O-bis (trimethylsilyl)trifluoroacetamide with trimethylchlorosilane (BSTFA with 1% TMCS, Thermo Scientific) for 1 h at 37 °C. The sample was allowed to equilibrate at room temperature for 1 h before 1 μL was injected onto the GC column using a hot needle technique. A spitless injection was performed for each sample.

##### 2.3.2.2 Instrument parameters

The GC–MS system comprised an AOC6000 autosampler and 2030 Shimadzu gas chromatograph coupled to a TQ8050NX triple quadrupole mass spectrometer (Shimadzu, Japan). The mass spectrometer was tuned according to the manufacturer's recommendations using tris-(perfluorobutyl)-amine (CF43). GC–MS analysis was performed on a 30 m Agilent DB-5 column with a 0.25 mm internal diameter column and 1 μm film thickness. The injection temperature (inlet) was set at 280 °C, the MS transfer line was set at 280 °C, and the ion source was adjusted to 200 °C. Helium was used as the carrier gas at a flow rate of 1 mL/min. The analysis of derivatized samples was performed under the following oven temperature program: 100 °C start temperature, hold for 4 min, followed by a 10 °C min^−1^ oven temperature ramp to 320 °C with a following final hold for 11 min. The mass spectrometer was operated in electron ionization mode with a scan range of 45–500 *m*/*z* at a 2000 scan speed.

The semi-targeted central carbon metabolites and their mass isotopologues were integrated in the DExSI software (version 3.5) [35]. Each peak integration was visually validated and manually corrected where required. The DExSI output for each compound was the fractional labelling value of the total compound pool corrected for the natural isotopic background abundance.

### 2.4 HSC activation and collagen secretion

LX-2 cells were seeded overnight, serum starved for 24 h then incubated in high glucose DMEM, supplemented with 1 μM l-carnitine, 2% BSA conjugated to either 0, 0.05, 0.5 or 1 mM palmitate, or 250 μM of palmitate and 250 μM of oleate (total fatty acid concentration = 0.5 mM), with or without 5 ng/mL TGF-β1 and/or 4.84 μM Lipofermata (Cayman Chemical, Cat: 25869).

For human liver secretion medium studies, following 24 h of serum starvation, LX-2 cells were cultured in 50% MEM and 50% human liver secretion medium, supplemented with 1 μM l-carnitine, and 2% BSA conjugated to 500 μM palmitic acid for 24 h. The incubation medium was collected and centrifuged at 1,000 g for 10 min and collagen secretion was assessed using human pro-collagen type 1 kit (Revvity, Cat: 63ADK014PEG) according to manufacturer's instructions. The cells were collected for qRT-PCR assessment.

### 2.5 Lipidomic analysis

The LX-2 lipidome was evaluated as previously described with few exceptions [30]. Briefly, cells were homogenised in 600 μL PBS (Gibco) with Zirconium Oxide beads and 10 μL Splash Mix II LIPIDOMIX Mass Spec Standard (330709W, Avanti Polar Lipids Inc, USA) and 215 μL of methanol. Lipids were extracted by the addition of 750 μL of methyl-Tert-Butyl Ether, and shaken for 30 min. Following this, 187.5 μL of MS grade H_2_O was added, samples were incubated for 10 min, centrifuged at 1000 x g for 20 min and the supernatant was collected and dried (Eppendorf Concentrator Plus, Germany). Samples were resuspended in 100 μL of chloroform: methanol (1:9, v:v), transferred into glass vials for lipidomic analysis by ultrahigh performance liquid chromatography coupled to tandem mass spectrometry employing a Vanquish UHPLC linked to an Orbitrap Fusion Lumos mass spectrometer (Thermo Fisher Scientific). MS data was processed using MS DIAL (Version 4.92). Relative quantification of lipid species was achieved by comparison of the LC peak areas of identified lipids against those of the corresponding internal lipid standard and the resultant ratio of peak area was normalized to protein content and visualised in R.

### 2.6 RNA extraction and real time polymerase chain reaction

RNA was isolated from LX-2 cells using TRI-Reagent (Sigma–Aldrich, Australia), DNA was removed with DNAse (Ambion DNA free kit, Thermo Fisher, Australia) and cDNA generated by reverse transcription cDNA with iSCRIPT Reverse Transcriptase (Invitrogen, USA) as per the manufacturer's instructions. Gene expression was assessed by Real-time PCR using the SYBR Green PCR master mix (Quantinova® SYBR Green PCR kit, QIAGEN, Germany). Expression was determined using a CFX Connect™ Real-Time PCR Detection System (Biorad, USA). The mRNA content within LX-2 cells were quantified by the 2−ΔΔCT method and normalised to *GAPDH or HPRT* [30]. The primer sequences are listed in Supplementary Table 1.

### 2.7 Data mining

Protein abundance of enzymatic regulators of pyruvate and phosphoenolpyruvate metabolism in murine liver, hepatocytes and HSCs were datamined from [36].

### 2.8 Statistical analysis

Statistical analyses were performed using GraphPad Prism (version 10) or R (version 4.3.2). Data was assessed for normal distribution using D'Agostino-Pearson tests. Statistical analyses included unpaired two-tailed Student's t-test, and one-way or two-way analysis of variance (ANOVA) for normally distributed data, where appropriate. Mann–Whitney tests were performed for data that was not normally distributed. Means were compared using Holm's Sidak post-hoc test where appropriate. Categorical data was assessed by Fisher's exact test. Human plasma biochemistries are reported as mean ± SD, all other data are shown as mean ± SEM, with statistical significance set as p < 0.05 or adjusted p-value <0.05.

## 3 Results

### 3.1 Upregulation of lipid metabolism pathways in HSCs in people with MASH

**Figure 1:**
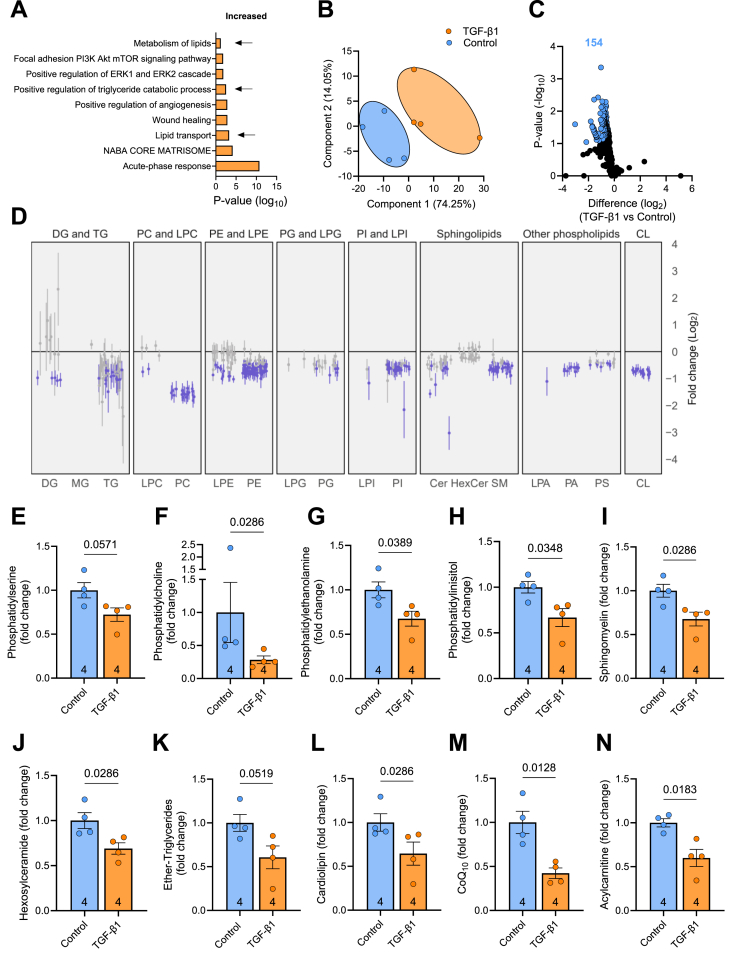
**Remodelling of the lipidome in activated LX-2 hepatic stellate cells. (A)** Metascape analysis of upregulated genes in hepatic stellate cells derived from livers of patients with MASH. **(B)** Principal component analysis of the lipidome in LX-2 cells treated with or without TGF-β1. **(C**) Volcano plot showing individual lipids and **(D)** forest plot of the lipid types from LX-2 cells cultured in the absence or presence of TGF-β1. CL, Cardiolipin. Purple bars represent significantly reduced lipid species. **(E)** Mass spectrometry lipidomic assessment of phosphatidylserine, **(F)** phosphatidylcholine, **(G)** phosphatidylethanolamine**, (H)** phosphatidylinositol, **(I)** sphingomyelin, (**J)** hexosylceramide, **(K)** ether-triglyceride, **(L)** cardiolipin, **(M)** co-enzyme Q10, and **(N)** acylcarnitine. Significance set a P < 0.05 and assessed by two-way t-tests with Benjamini-Hochberg false discovery rate (adjusted p-value <0.05, panel C & D), unpaired t-test (panel G, H, K, M & N) or Mann Whitney test (panel E, F, I, J & L) as appropriate. N = 4/group and listed in each column. Data are presented as mean ± SEM.

To determine whether lipid metabolism is remodelled in HSCs with MASH, we performed Metascape pathway analysis on single cell RNA sequencing data of HSCs derived from persons with non-alcoholic steatohepatitis (NASH, ostensibly MASH) [34]. The gene set analysed included 169 genes that were increased and 291 genes that were decreased in HSCs from persons with NASH/MASH compared with healthy liver tissue (Padj. < 0.05 and log2FC > 1) [34]. Compared to healthy liver tissue, biological pathways that were reduced in persons with MASH included ‘response to growth factors or hormones’ and ‘response to xenobiotic stimulus’ (Supplementary Fig. 1A). HSCs from persons with MASH exhibited an increase in genes involved in the ‘acute-phase response’, the ‘core matrisome/ECM proteins’, and ‘complement system pathways’ (Figure 1A, Supplementary Fig. 1B). Several metabolic pathways were enriched in HSCs derived from persons with MASH, including ‘lipid transport’, ‘triglyceride catabolism’ and ‘lipid metabolism’. This suggests that HSCs may regulate components of lipid metabolism to support their fibrogenic functions.

### 3.2 TGF-β1 remodels the lipidome in LX-2 HSCs

To determine whether lipid metabolism is altered with HSC activation, LX-2 cells were treated with palmitate, without or with TGF-β1 for 2 h, and the cellular lipidome was assessed by mass spectrometry lipidomics. TGF-β1 induced marked effects on the lipidome as evidenced by clear clustering of the groups using principal component analysis (Figure 1B). TGF-β1 reduced the levels of 154 individual lipid species (Figure 1C) and the forest plot highlights reduced content of many lipid types (Figure 1D). There was no significant increase in any lipid with TGF-β1 treatment. TGF-β1 reduced several glycerophospholipids such as phosphatidylserine (p = 0.057), phosphatidylcholine, phosphatidylethanolamine and phosphatidylinositol, and tended to reduce phosphatidylglycerol (p = 0.11) and phosphatidic acid (p = 0.09) (Figure 1E–H & Supplementary Fig. 1C&D). There were no changes in lysophospholipids (Supplementary Fig. 1E–I), apart from a reduction in N-acyl lysophosphatidylethanolamine (Supplementary Fig. 1J). TGF-β1 reduced sphingomyelin and hexosylceramides, but not other sphingolipids such as ceramides or GM3 gangliosides (Figure 1I–J, Supplementary Fig. 1K-L). The glycerolipids were mostly unaffected by TGF-β1 administration (e.g., triacylglycerol, diacylglycerol; Supplementary Fig. 1M−N) except for ether-triglycerides, which were reduced (Figure 1K). Notably, there were marked reductions in cardiolipin (Figure 1L), which is enriched in the mitochondria; CoQ10 (Figure 1M), which is a component of the electron transport chain and participates in ATP production; and acylcarnitines (Figure 1N), which serve as carriers to transport activated long-chain fatty acids into the mitochondria for β-oxidation. Together, these data demonstrate that activation of HSCs by TGF-β1 alters the cellular lipidome with significant remodelling of lipids enriched in the mitochondria.

### 3.3 TGF-β1 remodels fatty acid metabolism in LX-2 hepatic stellate cells

**Figure 2:**
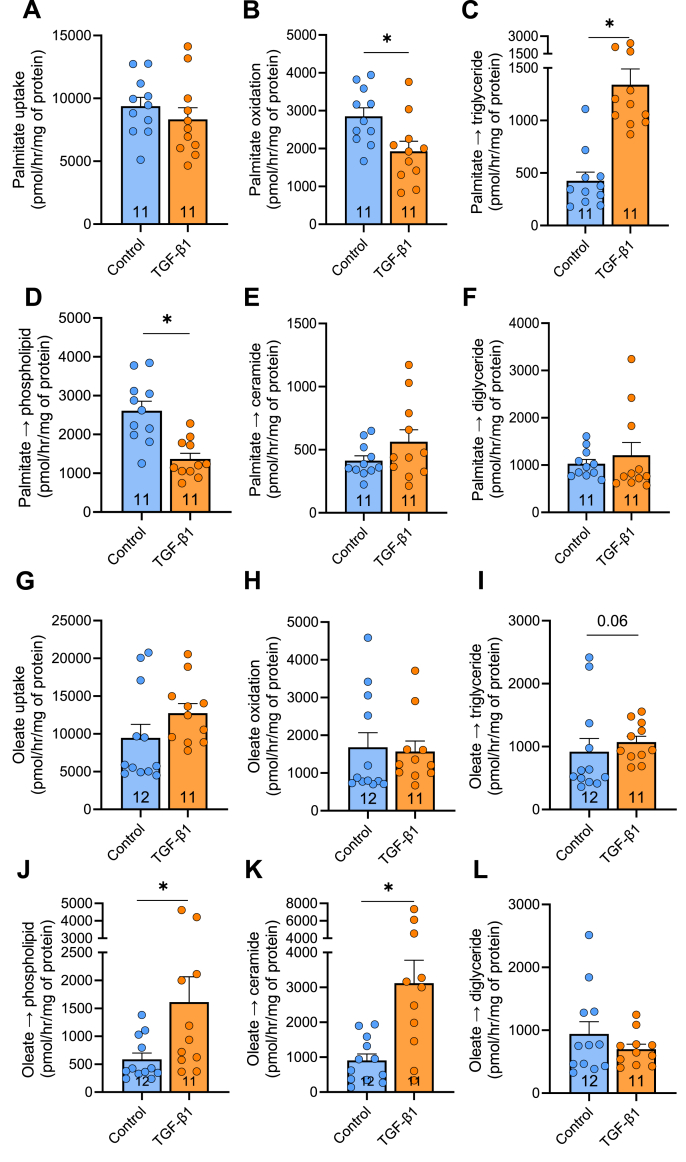
**Fatty acid metabolism in activated LX-2 hepatic stellate cells.** Radiometric tracing of palmitate metabolism in LX-2 cells showing **(A)** palmitate uptake, **(B)** palmitate oxidation **(C)** triglyceride synthesis, **(D)** diglyceride synthesis, **(E)** ceramide synthesis, and **(F)** phospholipid synthesis. **(G)** Radiometric tracing of oleate metabolism in LX-2 cells showing oleate showing palmitate uptake, **(H)** oxidation, **(I)** triglyceride synthesis, **(J)** phospholipid synthesis, **(K)** ceramide synthesis, and **(L)** diglyceride synthesis. ∗P < 0.05. Significance set a P < 0.05 and assessed by unpaired t-test (Panel A, B, E, F, & K) or Mann Whitney test (Panel C, D, G-J, L), with n = 11–12/group listed in each column. Data are presented as mean ± SEM.

In light of the significant lipidomic remodelling in activated HSCs, particularly in mitochondrial lipids, and previous studies highlighting the importance of CPT1 and fatty acid oxidation in HSC activation [27], we next assessed fatty acid metabolism using ^14^C-palmitate tracing in LX-2 cells with exposure to TGF-β1. TGF-β1 did not impact palmitate uptake (Figure 2A), whereas palmitate oxidation was decreased by 32% (Figure 2B). Palmitate incorporation into triglycerides was increased (312%, Figure 2C) and decreased in phospholipids (52%, Figure 2D), while incorporation into diglycerides and ceramides was not different when compared to vehicle control (Figure 2E&F).

Plasma contains a variety of fatty acids, such that about 35% are saturated and 65% are unsaturated, and different fatty acids can impart specific effects, or even opposing actions, on cellular functions. Accordingly, we next assessed oleate metabolism in LX-2 cells using ^14^C-oleate tracers. TGF-β1 did not impact oleate uptake or oxidation (Figure 2G,H). There was a trend for increased incorporation into triglycerides (p = 0.06, Figure 2I) but substantially increased incorporation into phospholipids (272%) and ceramides (∼326%) (Figure 2J,K). Oleate incorporation into diglycerides was not different between control and TGF-β1 treated cells (Figure 2L). Taken together, HSCs can readily transport and utilise fatty acids for ATP production and TGF-β1 mediated partitioning of fatty acids to oxidation and/or specific lipids is dictated by the type of fatty acid.

**Figure 3:**
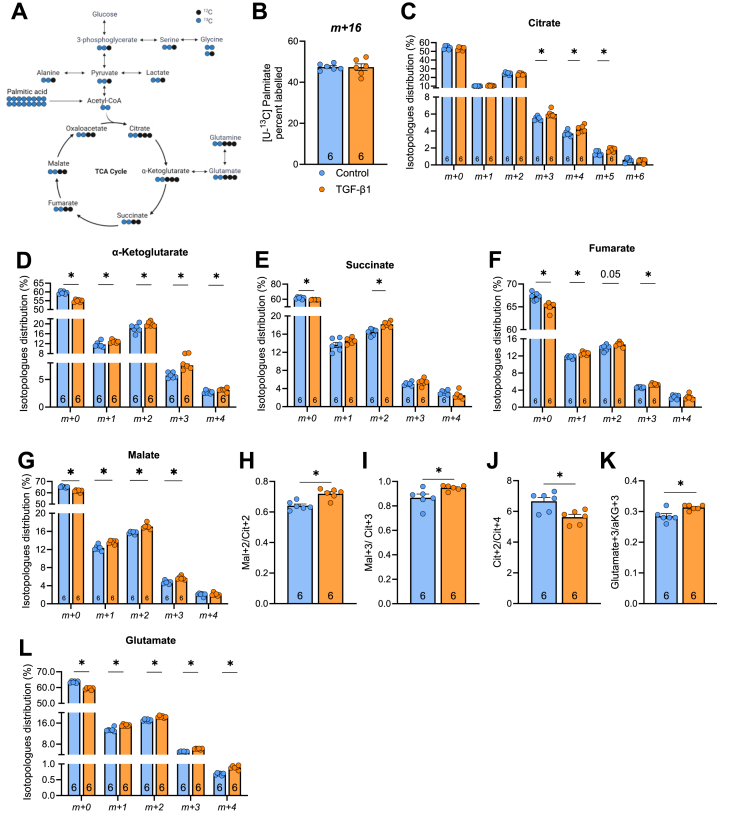
**^13^C-palmitate tracing in LX-2 cells reveals changes in TCA cycle labelling with exposure to TGF-β1. (A)** Schematic of fatty acid derived carbons into various metabolites. LX-2 cells were treated without (Control) or with TGF-β1. **(B)**^13^C palmitate enrichment and ^13^C incorporation into **(C)** citrate, **(D)** α-ketoglutarate, **(E**) succinate, **(F)** fumarate, **(G)** malate. **(H)** The mal+2/cit+2 and **(I)** mal+3/cit+3, and **(J)** cit+2/cit+4 ratio derived from [U-^13^C]palmitate. **(K)** Ratio of carbon labelling from α-ketoglutarate (m+3) to glutamate (m+3), and **(L)**^13^C incorporation into glutamate. Significance set a P < 0.05 and assessed by unpaired t-test (Panel B, D, & G-L) or Mann Whitney test (Panel C-F). N = 6/group and listed in each column. Data are presented as mean ± SEM.

To further explore changes in fatty acid metabolism, we utilised ^13^C-palmitate tracing in LX-2 cells to measure ^13^C incorporation into individual metabolites (Figure 3A). ^13^C-palmitate enrichment was similar between control and TGF-β1 treated groups (Figure 3B) and cell viability was similar between treatments (control: 87.0 ± 0.6%, TGF-β1: 87.4 ± 0.6%, palmitate: 87.6 ± 1.5%, TGF-β1 + palmitate: 86.2 ± 1.1%). TGF-β1 increased the enrichment of labelled carbons into all TCA cycle intermediates including citrate, αKG, succinate (*m*+2), fumarate, and malate (Figure 3C–G). The malate/citrate ratio, which is indicative of increased canonical TCA cycle flux [37], was increased with exposure to TGF-β1 (Figure 3H,I). Despite this, there was a reduction in the citrate m+2/citrate m+4 ratio (Figure 3J), reflecting reduced citrate regeneration through the oxidative TCA cycle [37]. This indicates that less fatty acid derived carbons are contributing to citrate regeneration and are likely exported out of the TCA cycle.

An interesting observation from these experiments was the enrichment of m+3, m+4 and m+5 isotopomers of citrate and αKG and no differences in m+3 and m+4 isotopomers of succinate (Figure 3D), which suggests the likelihood of increased flux from citrate to αKG then export into glutamate in TGF-β1 treated HSCs. Indeed, the enrichment of carbon labelling into glutamate and the ratio of carbon labelling from αKG (*m+3*) to glutamate (*m+3*), which partially represents the export from the TCA cycle, was increased with TGF-β1 compared to control (Figure 3K&I). Together, these experiments show that chronic TGF-β1-mediated HSC activation is associated with increased labelling of TCA cycle intermediates, reduced citrate regeneration and a modest flux of palmitate-derived carbon into glutamate.

### 3.4 Effect of palmitate on HSC activation and collagen production and secretion

**Figure 4:**
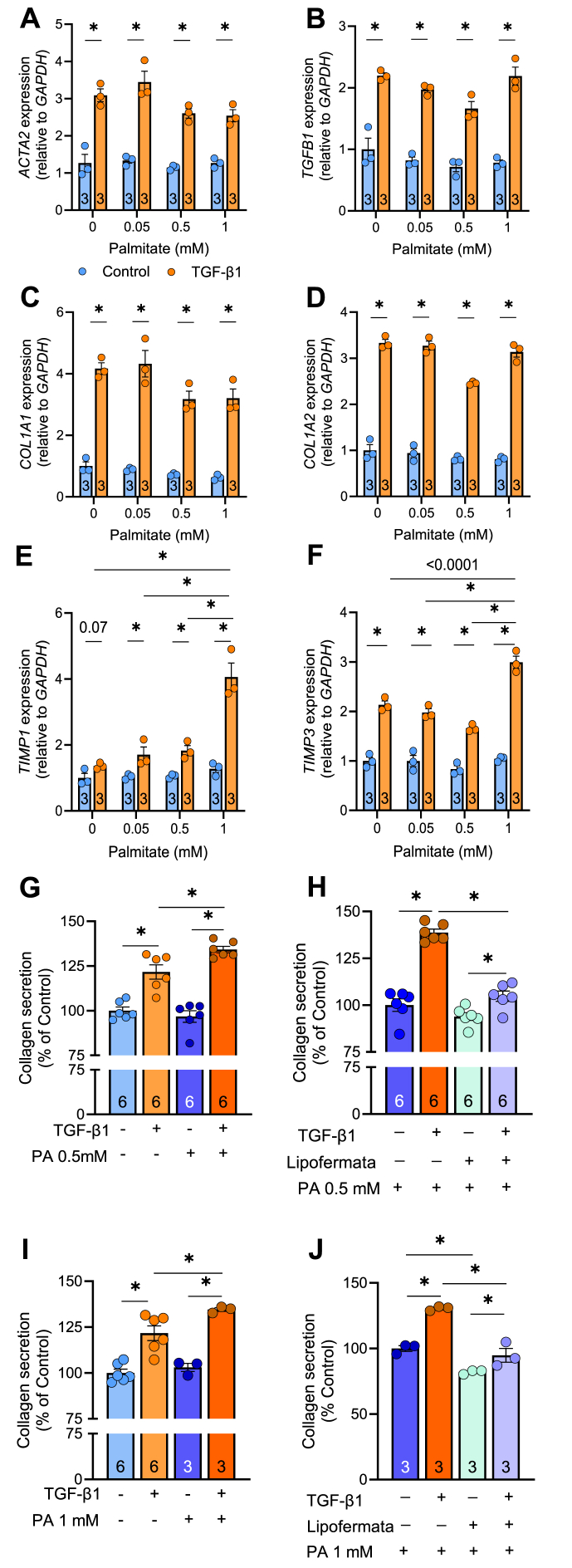
**Effect of palmitate on hepatic stellate cell activation.** LX-2 cells were cultured without (Control) or with TGF-β1 and varying concentrations of palmitate. mRNA expression of **(A)***ACTA2*, **(B)***TGFB1*, **(C**) *COL1A1*, **(D)***COL1A2*, **(E)***TIMP1*, and **(F)***TIMP3*. **(G)** Collagen secretion in the absence or presence of palmitate (0.5 mM) and TGF-β1 and, **(H)** in the presence of Lipofermata. **(I)** Collagen secretion in the absence or presence of palmitate (1 mM) and TGF-β1 and, **(J)** in the presence of Lipofermata. Significance set a P < 0.05 and assessed by two-way ANOVA with Holms-Sidak multiple comparisons (Panel A-F, I-J) or one-way ANOVA with Holms-Sidak multiple comparisons (Panel G-H). N = 3–6/group and listed in each column. Data are presented as mean ± SEM.

MASH is associated with increased fatty acid availability to the liver parenchyma [38]. Accordingly, we next asked whether increasing fatty acid availability was sufficient to activate HSCs. LX-2 cells were cultured with increasing concentrations of palmitate, with or without TGF-β1 for 24 h. As expected, TGF-β1 increased the expression of pro-fibrogenic genes by 2-3-fold, whereas palmitate did not increase *ACTA2, TGFB1, COL1A1, COL1A3, TIMP1 or TIMP3* mRNA expression (Figure 4A–F). Palmitate did not augment the expression of pro-fibrotic genes with concurrent TGF-β1 treatment, except for *TIMP1* and *TIMP3*, which were increased in the presence of 1 mM palmitate, but not in cells exposed to lower palmitate concentrations (ranging from 0 to 0.5 mM) (Figure 4E–F).

Extending on these molecular measures, proCol1a1 secretion was assessed as a functional readout of HSC activation. TGF-β1 increased collagen secretion, as expected (Figure 4G). Palmitate did not impact collagen secretion, but amplified TGF-β1 induced collagen secretion (Figure 4G). We next asked whether inhibition of fatty acid uptake could suppress collagen secretion in the context of increased fatty acid availability. Lipofermata, an inhibitor of the fatty acid transporter FATP2, did not impact collagen secretion under basal conditions, however, in the presence of TGF-β1, Lipofermata attenuated TGF-β1-induced collagen secretion by 34%, levels that were indistinguishable from control (Figure 4H). These findings were recapitulated at higher palmitate concentrations (i.e., 1 mM; Figure 4I–J).

We next assessed whether a mixture of fatty acids, in this case palmitate and oleate, could also enhance TGF-β1-mediated collagen secretion. As expected, TGF-β1 increased collagen secretion in cells treated with or without the fatty acid mixture. However, the presence of oleate (Supplementary Fig. 2A) prevented the palmitate-mediated amplification of TGF-β1-induced collagen secretion (Figure 4G–J). Inhibition of fatty acid uptake with Lipofermata attenuated TGF-β1-induced collagen secretion to levels indistinguishable from unstimulated LX-2 cells (Supplementary Fig. 2A). Together, these data indicate that palmitate does not induce molecular reprogramming towards a pro-fibrogenic state, rather palmitate, in the absence of oleate, enhances TGF-β1-mediated collagen secretion. This raises the possibility that (1) palmitate allows for re-routing of other metabolites for collagen synthesis, and/or that (2) palmitate provides a carbon source for amino acid production to facilitate collagen synthesis and secretion.

### 3.5 Glucose incorporation into select intracellular amino acids is reduced with palmitate

**Figure 5:**
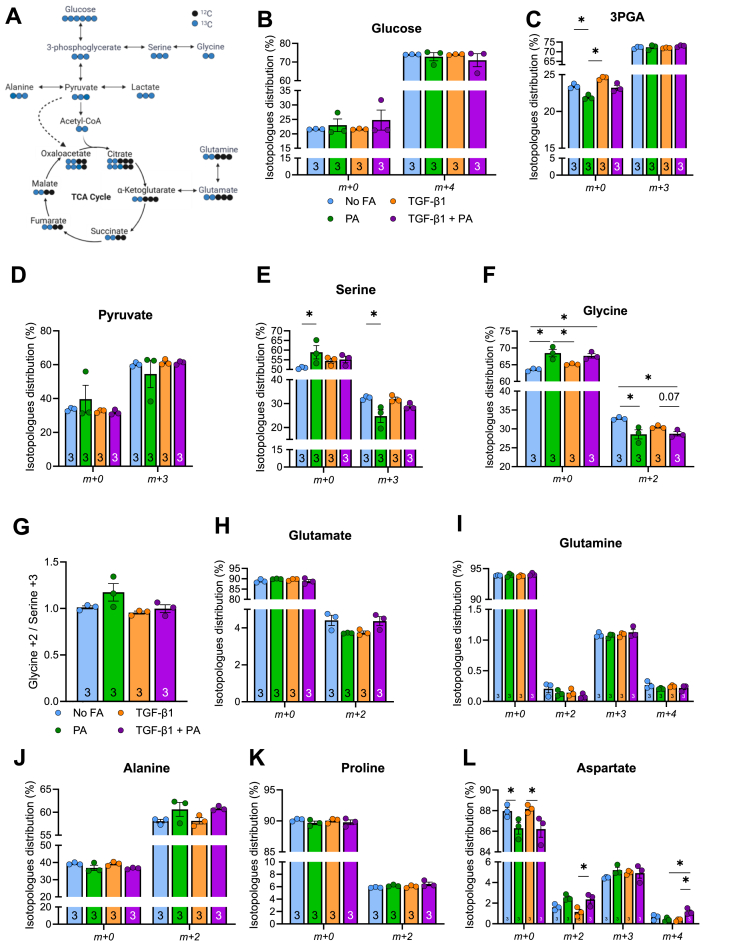
**Glucose-derived carbon incorporation into collagen precursor amino acids with palmitate exposure in LX-2 cells. (A)** Schematic representation of glucose flux towards glycine. **(B**–**L)** LX-2 cells were treated with TGF-β1 and/or palmitate and ^13^C-glucose. Percent labelling in **(B)** glucose, **(C)** glycerol-3-phosphate, **(D)** pyruvate, **(E)** serine, **(F)** glycine. **(G)** Glycine/serine ratio. **(H)**^13^C-glucose derived carbon incorporation into glutamate, **(I)** glutamine, **(J)** alanine, **(K)** proline, and **(L)** aspartate. Significance set a P < 0.05 and data assessed by two-way ANOVA and Holms-Sidak multiple comparisons (Panel B-L). N = 3/group and listed in each column. Data are presented as mean ± SEM.

Accordingly, we next investigated whether palmitate availability diverts glucose-derived carbons towards the production of collagen amino acid precursors. LX-2 cells were incubated with TGF-β1 and/or 0.5 mM palmitate for 2 h and the fate of isotopic U-^13^C-glucose was traced for a further 2 h. The theoretical carbon fate of glucose is depicted in Figure 5A. There were no differences in the uptake of ^13^C-glucose or incorporation of glucose into glycolytic intermediates 3PGA and pyruvate with or without TGF-β1 and/or 0.5 mM palmitate (Figure 5B–D, e.g. no change in m+3 labelling). Palmitate decreased glucose labelling of serine (*m+3*) and glycine (*m+2*) (Figure 5E,F), but no treatment group impacted the flux of serine to glycine (Figure 5G). Other amino acids that are critical for collagen synthesis including glutamate, glutamine, alanine, and proline were unaffected by TGF-β1 and/or palmitate treatment (Figure 5H-L). Palmitate did not alter glucose-derived carbons into aspartate, however, following the addition of TGF-β1, *m+2* and *m+4* isotopologues of aspartate were increased with palmitate exposure (Figure 5L). Together, these findings suggest that diversion of glucose carbons to serine and glycine, but not other amino acids, is mildly attenuated with palmitate exposure and largely unaffected by TGF-β1.

### 3.6 Carbons contained within palmitate are used for amino acid synthesis in activated HSCs

**Figure 6:**
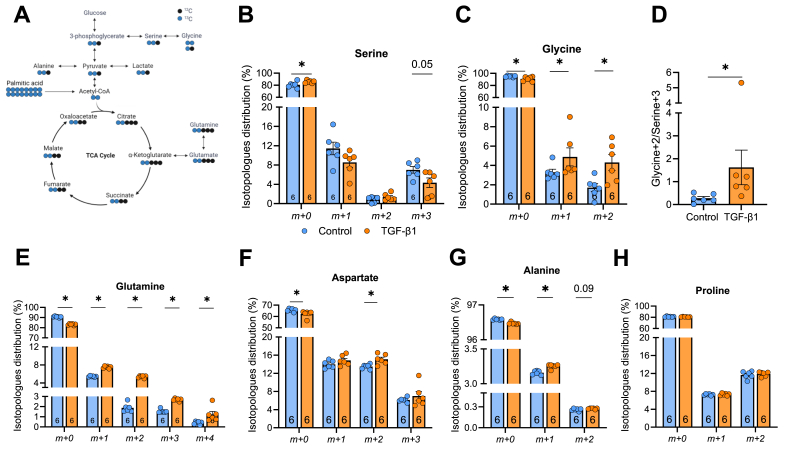
**Palmitate-derived carbon incorporation into collagen precursor amino acids in LX-2 cells.** LX-2 cells were cultured in the absence (Control) or presence of TGF-β1, and 13C-labelled palmitate. **(A)** Schematic representation of palmitate flux to glycine. Percent labelling of palmitate-derived ^13^C into **(B)** serine, **(C)** glycine, **(D**) glycine (m+2)/serine (m+3) ratio, **(E)** glutamine, **(F)** aspartate, **(G)** alanine, **(H)** proline. Significance set a P < 0.05 and assessed by unpaired t-test (Panel B-H). N = 6/group and listed in each column. Data are presented as mean ± SEM.

We next assessed whether palmitate-derived carbons could be used to synthesize amino acids (Figure 6A). With prolonged (48 h) exposure to TGF-β1 in the presence of ^13^C palmitate, ^13^C incorporation into serine was reduced (*m*+3, p = 0.05) with TGF-β1 (Figure 6B), however, this coincided with a concomitant increase in glycine isotopologues (48% m+1, 251% m+2, Figure 6C). The palmitate-derived carbon labelling accounted for 9.2% of the total labelled glycine pool in TGF-β1 treated HSCs compared with 5% in control HSCs (Figure 6C), and this was likely derived from increased flux from serine (Figure 6D). Prolonged TGF-β1 treatment increased the labelled glutamine (81%), and marginally increased glutamate (15%), aspartate (10%) and alanine (2%) labelling (Figure 3, Figure 6E-G). Proline labelling was not different between groups (Figure 6H). Together, these findings show that carbons derived from palmitate are used for the synthesis of collagen precursor amino acids, that this process is increased in activated compared with quiescent HSCs, and that this process is more pronounced with prolonged exposure to TGF-β1.

### 3.7 Liver-secreted factors from patients with MASLD enhance palmitate-mediated HSC activation

**Figure 7:**
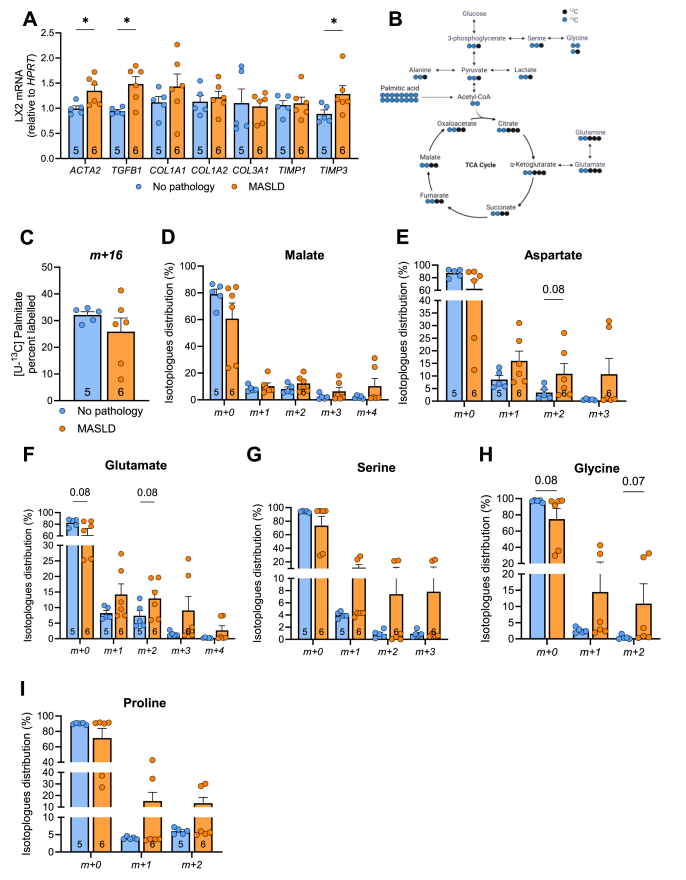
**MASLD remodelled human liver secreted factors regulates of HSC activation and palmitate incorporation into amino acids.** LX2 cells were cultured in the presence of 0.5 mM palmitate and liver secreted factors from individuals from humans with MASLD or no liver pathology, followed by assessment of HSC activation, fibrogenesis, and isotopic tracing. **(A)** mRNA expression of markers of HSC activation and fibrogenesis. **(B)** Schematic of fatty acid derived carbons into various metabolites. **(C)**^13^C palmitate enrichment and ^13^C incorporation into **(D)** malate, **(E**) aspartate, **(F)** glutamate, **(G)** serine, **(G)** glycine, **(G)** proline. Significance set a P < 0.05 and data assessed by Mann Whitney test (Panel A, C-I). N = 5–6/group and listed in each column. Data are presented as mean ± SEM.

To recapitulate the extracellular milieu that HSCs are exposed to *in vivo*, LX-2 cells were cultured in the presence of palmitate and liver-secreted factors derived from humans with obesity, with or without MASLD [31] (Supplementary Fig. S3A), followed by assessment of HSC activation and palmitate metabolism. The liver secretion medium contains proteins [31], lipids, and metabolites [39] that have previously been described by our group. Liver slice mass and viability, and clinical biochemistries were not different between groups (Fig. S3B&C, Supplementary Table 2). In the presence of liver secreted factors from patients with MASLD, palmitate increased the expression of HSC activation genes *ACTA2* and *TGFB1*, and *TIMP3* in LX-2 cells. The mRNA content of *COL1A1, COL1A2, COL3A1* and *TIMP1* were not different compared to LX-2 cells incubated in palmitate and liver secreted factors from patients with No pathology (Figure 7A).

To determine whether the milieu of liver secreted factors influences metabolism in HSCs, we replicated these experiments (i.e., cultured LX-2 cells in secreted factors from patients with MASLD or No pathology) and performed ^13^C palmitate tracing (Figure 7B). MASLD-liver secreted factors did not influence palmitate uptake or palmitate-derived carbon incorporation into malate isotopologues (Figure 7C,D), but tended to increase carbon incorporation into aspartate and glutamate (m+2) (Figure 7E–F). There was no difference between groups for carbon incorporation into serine (Figure 7G). MASLD-secreted factors tended to increase palmitate incorporation into the m+2 glycine isotopomer (p = 0.07, Figure 7H) and no differences were observed with proline labelling between groups (Figure 7I). Together these findings show that MASLD-secreted factors can activate HSCs, and this is associated with an increased incorporation of palmitate into several amino acids.

## 4 Discussion

Aberrant lipid metabolism in hepatocytes is a well-established feature of MASLD [40,41], however, the influence of fatty acid availability and metabolism on HSC activation and fibrogenesis remains poorly characterized. In this study, we demonstrate that TGF-β1-induced activation of HSCs is accompanied by reprogramming of fatty acid utilization and remodelling of the cellular lipidome. Exogenous fatty acids did not induce HSC activation. Rather, palmitate enhanced TGF-β1-driven flux of palmitate-derived carbon into glycine biosynthesis and subsequent collagen production, while blocking fatty acid uptake prevented TGF-β1 mediated collagen secretion. Notably, equimolar concentrations of oleate blocked palmitate-mediated alterations in collagen production. Further, liver secreted factors from patients with MASLD activated HSCs to a greater extent than factors from livers with no pathology, which was associated with a subtle increase in glycine synthesis from palmitate-derived carbons. These findings suggest that activated HSCs undergo metabolic adaptations that prioritise anabolic pathways essential for collagen synthesis.

Reprogramming of metabolism occurs during HSC activation, with important roles described for glucose, lactate and glutamine in meeting the energy demands of transdifferentiation [42]. In contrast, the changes in lipid metabolism during HSC activation are not well described, particularly the role of exogenous non-esterified fatty acids. A hallmark of HSC activation is the rapid depletion of intracellular LDs (Figure 1) [12,43]. While the LDs in HSCs are characterized for their abundance of retinyl esters, they also contain significant TG stores (∼30% by mass) [44], indicating that they are likely to be important substrates during HSC activation [23,45]. It is also likely that lipophagy, which is the autophagic degradation of intracellular LD, is important for HSC activation. Loss of autophagic function in cultured mouse HSCs and in mice following injury reduced fibrogenesis and matrix accumulation, an effect partially overcome by providing exogenous fatty acid in the culture medium [46]. To this point, the present studies examined the fate of exogenous fatty acids and lipidomic remodelling with TGF-β1 induced HSC activation. Our studies using ^14^C-palmitate and ^14^C-oleate show that fatty acid uptake is not increased with HSC activation, rather fatty acid partitioning is altered. Oleate is preferentially channelled towards phospholipid and ceramide production, while less palmitate is directed to oxidation for ATP production. This latter effect could be due to diversion of TCA cycle intermediates away from oxidative phosphorylation (discussed below) and/or re-direction of palmitate to other fates such as triglyceride storage. The latter possibility aligns with observations in other cell types that fatty acids taken up from the extracellular environment first traverse the LD before their eventual oxidation [47,48]. Detailed tracing studies are required to test this possibility in HSCs.

Transdifferentiation of HSCs into an activated myofibroblast-like state is characterized by cell proliferation and increased production and secretion of collagens and other ECM proteins. The predominant ECM proteins involved in fibrogenesis are members of the collagen family. Collagens are characterized by repeating amino acid sequences, primarily glycine-proline-X and glycine-X-hydroxyproline motifs, where "X" represents any amino acid except glycine or (hydroxy)proline; commonly alanine, glutamate, aspartate, or serine. These specific sequence patterns are essential for proper folding and the formation of stable collagen fibers [15,16]. Glycine represents up to 33% [17] and proline constitutes up to 22% [49] of all amino acids present in collagen. Glucose derived carbons represent less than 10% of the proline pool in fibroblast cell lines, with glutamine dominating as the substrate for proline biosynthesis [49,50], likely explaining the lack of changes observed for glucose and fatty acid derived proline biosynthesis in this study. Although the contribution of carbons derived from various nutrients to glycine production is not described in HSCs, evidence from isotopic tracing studies in cancer associated fibroblasts and TGF-β1 stimulated fibroblasts [49,51] show that glucose-derived carbon incorporation into glycine and collagen is increased, suggesting that glucose is the major substrate for this process.

Activated HSCs upregulate glucose transport and glycolytic flux for ATP synthesis and for the provision of carbon for serine-glycine biosynthesis and eventual incorporation into collagen [41]. Notably, these previous studies were conducted in the absence of exogenous fatty acids, whereas the present studies focussed on the effect of palmitate on HSC metabolism and activation given that the liver parenchyma is exposed to a lipid-rich environment in MASLD. Under these experimental conditions, we define a role for palmitate in reducing the flux of glucose derived carbons to serine and glycine production, which was associated with a concomitant increase in palmitate-derived carbons towards glycine synthesis and collagen production. The reduction in carbon labelling into serine was anticipated based on previous observations in HeLa cells where the serine output rate was 7.3-fold greater than glycine output [52], likely indicative of carbons from serine being rapidly donated to glycine [41]. This process would require β-oxidation of palmitate to acetyl-CoA and flux through the TCA cycle, given that acetyl-CoA cannot be used to produce pyruvate, but TCA cycle intermediates (e.g. malate and α-ketoglutarate) can enter gluconeogenic pathways to form 3-phosphoglycerate for serine then glycine biosynthesis [53]. This paradigm is supported by isotopic tracing data showing enrichment of palmitate-derived carbons in malate and α-ketoglutarate, a decline in citrate regeneration which is indicative of export prior to oxaloacetate conversion to citrate, and the expression of genes encoding the regulatory enzymes of gluconeogenesis in HSCs (Supplementary Fig. 4). In support of this premise, silencing of CPT1A in LX-2 HSCs and in mice reduced HSC activation and fibrosis, despite no change in glycolytic flux [27]. Moreover, we observed increased incorporation of palmitate-derived carbons into glutamine and alanine, which are the other major components of collagen. Glutamine is presumably produced via reductive amination of α-ketoglutarate to glutamate, then glutamate to glutamine (see Figure 6A), while alanine can be derived from malate/oxaloacetate conversion to PEP, conversion to pyruvate, and transamination of pyruvate to alanine. Thus, instead of primarily serving as a fuel for increased ATP production in activated HSCs, palmitate is metabolised to support anaplerotic pathways that supply amino acids needed for collagen synthesis. However, this may be dependent on enhanced palmitate delivery towards the liver.

Dietary lipids are primarily absorbed through the mesenteric lymphatics and transported towards the bloodstream. Palmitate levels in mesenteric lymph of lean mice is 31-fold higher than oleate [54]. In obese mice, mesenteric lymphatics become ‘leaky’ and lymph fluid spills into mesenteric adipose tissue [55], where it may be taken up by adipocytes and/or transported through the portal circulation, thereby exposing the liver to increased palmitate levels. This represents a biologically plausible mechanism whereby HSCs are exposed to more palmitate *in vivo*. We show that palmitate-mediated amplification of TGF-β1 induced collagen secretion is prevented by co-treatment of an equimolar concentration of oleate (Supplementary Fig. 2). This limited fibrogenic response in cultured cells is most likely a reflection of the palmitate: oleate ratio, which would not be representative of the hepatic interstitial palmitate: oleate ratio for the reasons described above. Future studies should profile the portal lipidome and hepatic interstitial fluid lipidome in lean and obese states to better understand the composition of factors that the liver is exposed to in obesity.

There are several limitations to this study. We have used palmitate (C16:0) and oleate (C18:1) in our culture medium and it is likely that other fatty acids of varying chain lengths and desaturation will induce different effects. Extending on this point, HSCs are exposed to a milieu of factors *in vivo* including TGF-β, PDGF, and inflammatory cytokines, among others, that drive signalling cascades leading to transdifferentiation [56]. While we endeavoured to mimic the HSC microenvironment by exposing them to liver secreted factors from patients without and with MASLD, a variety of factors including, but not limited to, MASLD endotypes [57] and genetic polymorphisms may influence the liver secretome. Additionally, these studies relied on cultured cells and future studies will need to validate these findings *in vivo*. Evidently, this is technically difficult given the heterogeneity of cell types in the liver which precludes direct assessment of HSCs metabolism and an inability to obtain sufficient primary HSCs using current isolation protocols.

In conclusion, we used radiolabelled and stable palmitate tracers to demonstrate that TGF-β1 induces significant remodelling of fatty acid metabolism in HSCs. LX-2 cells reprogram their metabolism in the presence of exogenous palmitate by increasing the provision of fatty acid-derived carbons into collagen, which are subsequently secreted. These studies provide a mechanistic advance in the understanding of fibrometabolism, and future studies should determine whether such remodelling of palmitate metabolism contributes to fibrogenesis in MASLD.

## CRediT authorship contribution statement

**William De Nardo:** Conceptualization, Data curation, Formal analysis, Investigation, Methodology, Project administration, Resources, Software, Supervision, Validation, Visualization, Writing – original draft, Writing – review & editing. **Jacqueline Bayliss:** Investigation, Methodology, Resources. **Sheik Nadeem Elahee Doomun:** Investigation, Methodology, Writing – review & editing. **Olivia Lee:** Formal analysis, Investigation, Methodology, Visualization, Writing – review & editing. **Paula M. Miotto:** Investigation, Methodology, Writing – review & editing. **Natasha D. Suriani:** Investigation, Methodology. **Shuai Nie:** Data curation, Methodology. **Michael Leeming:** Methodology, Project administration, Supervision. **Diego A. Miranda:** Conceptualization, Supervision, Writing – review & editing. **David P. De Souza:** Methodology, Resources, Software, Supervision, Writing – review & editing. **Matthew J. Watt:** Conceptualization, Data curation, Formal analysis, Funding acquisition, Project administration, Resources, Software, Supervision, Writing – original draft, Writing – review & editing.

## Financial support

This work was supported by funding from 10.13039/100005564Gilead Sciences, United States and the 10.13039/100009648National Health and Medical Research Council of Australia, Australia (NHMRC, Grant ID: 2020078). WDN was supported by The University of Melbourne, Australia Research scholarship. OL and NDS are supported by Australian Government Research Training Program (RTP) Scholarships. PMM is supported by an NHMRC EL1 Investigator Fellowship (ID: 2018187).

## Declaration of competing interest

The authors declare the following financial interests/personal relationships which may be considered as potential competing interests: Matthew Watt reports financial support was provided by Gilead Sciences. Matthew Watt reports financial support was provided by National Health and Medical Research Council. Diego Miranda reports a relationship with Gilead Sciences that includes: employment. Matthew Watt reports a relationship with Gilead Sciences that includes: consulting or advisory. If there are other authors, they declare that they have no known competing financial interests or personal relationships that could have appeared to influence the work reported in this paper.

## Data Availability

Data will be made available on request.

## Footnotes

Appendix A Supplementary data to this article can be found online at https://doi.org/10.1016/j.molmet.2025.102309.
