# Supplementary material for: Effect of free fatty acids on TGF-β1 mediated fibrogenesis in hepatic stellate cells
Source: Mol Metab. 2025 Dec 17;104:102309. doi: 10.1016/j.molmet.2025.102309 (PMC12829130; doi:10.1016/j.molmet.2025.102309)
Supplement: Multimedia component 1 [file mmc1.docx]

**Supplementary Tables:**

**Supplementary Table 1: Primer list**

| **Genes** | **Forward Sequence** | **Reverse Sequence** |
| --- | --- | --- |
| ***ACTA2*** | CATCCTCATCCTCCCTTGAG | ATGAAGGATGGCTGGAACAG |
| ***COL1A1*** | GAACGCGTGTCATCCCTTGT | GAACGAGGTAGTCTTTCAGCAACA |
| ***COL1A2*** | GTGGTTACTACTGGATTGAC | CTGCCAGCATTGATAGTTTC |
| ***TGFB1*** | AACCCACAACGAAATCTATG | CTTTTAACTTGAGCCTCAGC |
| ***TIMP1*** | CACCTTATACCAGCGTTATG | TTTCCAGCAATGAGAAACTC |
| ***TIMP3*** | CATGTGCAGTACATCCATAC | AGGTGATACCGATAGTTCAG |
| ***HPRT*** | ATAAGCCAGACTTTGTTGG | ATAGGACTCCAGATGTTTCC |
| ***GAPDH*** | ATGACATCAAGAAGGTGGTG | CATACCAGGAAATGAGCTTG |

**Supplementary Table 2: Clinical biochemistries of humans with obesity, with or without MASLD.**

| **Parameter** | **No pathology (n=5)** | **MASLD (n=6)** | **P-value** |  |
| --- | --- | --- | --- | --- |
|  |  |  |  |  |
| **Age (years)** | 40.40 ± 11.70 | 40.17 ± 11.34 | 0.97 |  |
| **Gender (male, %)** | 1, 20.0% | 3, 50% | 0.45 |  |
| **Height (m)** | 1.70 ± 0.09 | 1.68 ± 0.11 | 0.83 |  |
| **T2D (n,%)** | 1, 20.0% | 3, 50% | 0.50 |  |
| **Hypertension (n,%)** | 1, 20.0% | 1, 16.6% | 0.99 |  |
| **Weight (kg)** | 124.72 ± 14.61 | 126.47 ± 21.48 | 0.88 |  |
| **BMI (kg/m^2^)** | 43.38 ± 5.20 | 44.63 ± 7.78 | 0.76 |  |
| **Urea (mmol/l)** | 4.36 ± 1.14 | 4.86 ± 1.73 | 1.00 |  |
| **Creatine (µmol/L)** | 75.00 ± 12.73 | 76.00 ± 10.62 | 0.89 |  |
| **Glucose (mmol/L)** | 5.80 ± 1.04 | 4.93 ± 0.64 | 0.15 |  |
| **Albumin (g/L)** | 36.80 ± 3.90 | 42.67 ± 5.05 | 0.06 |  |
| **Bilirubin (µmol/L)** | 8.60 ± 6.11 | 9.50 ± 3.83 | 0.78 |  |
| **ALT (U/L)** | 40.80 ± 44.90 | 62.33 ± 27.24 | 0.14 |  |
| **AST (U/L)** | 63.20 ± 82.80 | 41.83 ± 11.29 | 0.10 |  |
| **GGT (U/L)** | 33.80 ± 12.21 | 33.83 ± 16.31 | 0.99 |  |
| **ALP (U/L)** | 74.00 ± 17.25 | 74.50 ± 6.95 | 0.95 |  |
| **Cholesterol (mmol/l)** | 4.16 ± 0.93 | 4.32 ± 0.35 | 0.74 |  |
| **HDL (mmol/l)** | 1.01 ± 0.06 | 0.91 ± 0.08 | 0.05 |  |
| **LDL (mmol/l)** | 2.42 ± 0.88 | 2.60 ± 0.21 | 0.52 |  |
| **Triglycerides (mmol/l)** | 1.62 ± 0.54 | 1.78 ± 0.60 | 0.65 |  |
| **Vitamin B12 (pmol/L)** | 310.60 ± 104.45 | 485.67 ± 151.30 | 0.05 |  |
| **Iron (µmol/L)** | 12.80 ± 4.32 | 15.67 ± 5.16 | 0.34 |  |
| **Ferritin (ng/mL)** | 126.60 ± 156.27 | 267 ± 213.32 | 0.10 |  |
| **Vitamin D (nmol/L)** | 55.00 ± 26.37 | 58.67 ± 15.79 | 0.79 |  |
| **HbA1c (%)** | 5.48 ± 0.47 | 5.68 ± 0.37 | 0.45 |  |
| **C-peptide (nmol/L)** | 1.25 ± 0.48 | 1.09 ± 0.30 | 0.53 |  |
| **Insulin (mU/L)** | 16.50 ± 11.77 | 12.72 ± 7.63 | 0.56 |  |
| **HOMA2B** | 128.90 ± 86.30 | 138.22 ± 53.45 | 0.84 |  |
| **HOMA2S** | 74.42 ± 56.50 | 77.25 ± 34.92 | 0.93 |  |
| **HOMA2IR** | 2.14 ± 1.50 | 1.61 ± 0.96 | 0.52 |  |
| **Thyroid stimualting hormone (mU/L)** | 2.11 ± 1.01 | 2.25 ± 1.36 | 0.84 |  |
| **Haemoglobin (g/L)** | 137.40 ± 15.14 | 150.33 ± 14.76 | 0.19 |  |
| **White cell count (x 10^9^)** | 7.58 ± 2.25 | 7.35 ± 1.66 | 0.86 |  |
| **Platelet (10^9^/L)** | 308.60 ± 61.24 | 284.67 ± 68.32 | 0.56 |  |
| **MCV (fl)** | 88.40 ± 3.21 | 88.83 ± 3.31 | 0.83 |  |
| **MCH (pg)** | 28.40 ± 1.82 | 29.17 ± 1.83 | 0.51 |  |
| **MCHC (g/L)** | 319.00 ± 17.19 | 327.17 ± 8.38 | 0.37 |  |
| **Histology** |  |  |  |  |
| **Steatosis score** |  |  | <0.0001 |  |
| **0** | 5 (100%) | 0 (0%) |  |  |
| **1** | 0 (0%) | 1 (16.6%) |  |  |
| **2** | 0 (0%) | 5 (83.3%) |  |  |
| **3** | 0 (0%) | 0 (0%) |  |  |
| **Inflammation score** |  |  | <0.0001 |  |
| **0** | 5 (100%) | 0 (0%) |  |  |
| **1** | 0 (0%) | 3 (50%) |  |  |
| **2** | 0 (0%) | 3 (50%) |  |  |
| **3** | 0 (0%) | 0 (0%) |  |  |
| **Ballooning score** |  |  | 0.99 |  |
| **0** | 0 (0%) | 0 (0%) |  |  |
| **1** | 0 (0%) | 0 (0%) |  |  |
| **2** | 0 (0%) | 0 (0%) |  |  |
| **NAS score** |  |  | <0.0001 |  |
| **0** | 5 (100%) | 0 (0%) |  |  |
| **1** | 0 (0%) | 0 (0%) |  |  |
| **2** | 0 (0%) | 0 (0%) |  |  |
| **3-4** | 0 (0%) | 6 (100%) |  |  |
| **≥5** | 0 (0%) | 0 (0%) |  |  |
| **Fibrosis** |  |  | <0.0001 |  |
| **F0** | 5 (100%) | 0 (0%) |  |  |
| **F1** | 0 (0%) | 1 (16.6%) |  |  |
| **F2** | 0 (0%) | 5 (83.3%) |  |  |
| **F3** | 0 (0%) | 0 (0%) |  |  |
| **F4** | 0 (0%) | 0 (0%) |  |  |

ALP, alkaline phosphatase; ALT, alanine transferase; AST, aspartate transferase; BMI, body mass index; eGFR, estimated glomerular filtration rate; GGT, gamma-glutamyl transferase; Hb, hemoglobin; HbA1c, glycosylated haemoglobin; HDL, High-density lipoprotein; LDL, low-density lipoprotein; MCV, mean corpuscular volume; MCH, mean corpuscular hemoglobin; MCHC, mean corpuscular hemoglobin concentration; TSH, Thyroid-stimulating hormone; WCC, white cell count. Significant difference (p<0.05) compared to No pathology determined by either students t-test, Mann-whitney test, or fishers exact test, where appropriate.
