# Supplementary material for: Effect of free fatty acids on TGF-β1 mediated fibrogenesis in hepatic stellate cells
Source: Mol Metab. 2025 Dec 17;104:102309. doi: 10.1016/j.molmet.2025.102309 (PMC12829130; doi:10.1016/j.molmet.2025.102309)
Supplement: Multimedia component 2 [file mmc2.docx]

**
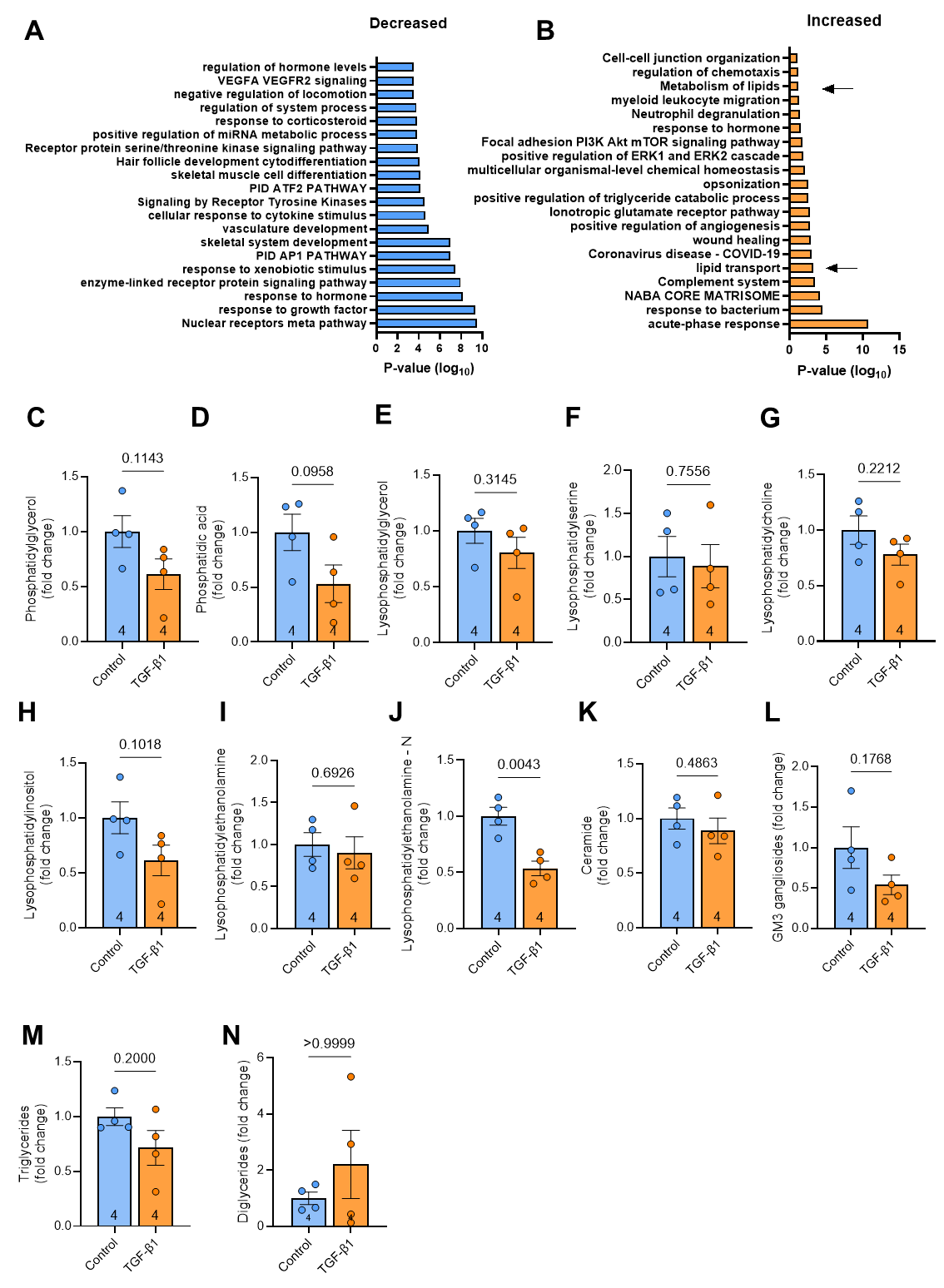
**

**Supplementary Figure 1. Remodelling of the lipidome in activated LX-2 hepatic stellate cells.**

**(A)** Metascape analysis of downregulated and, **(B)** upregulated genes in hepatic stellate cells derived from liver of patients with MASH. **(C-N**) Lipidomic assessment in LX-2 cells treated without (Control) or with TGF-β1, including **(C)** phosphatidylglycerol, **(D)** phosphatidic acid, **(E)** lysophosphatidylglycerol, **(F)** lysophosphatidylserine, **(G)** lysophosphatidylcholine, **(H)** lysophosphatidylinositol, **(I)** lysophosphatidylethanolamine, (**J)** lysophosphatidylethanolamine-N, **(K)** ceramide, **(L)** GM3 gangliosides, **(M)** triglycerides and **(N)** diglycerides. Significance set a P<0.05 and assessed by unpaired t-test or Mann Whitney test as appropriate. N=4/group and listed in each column. Data are presented as mean ± SEM.


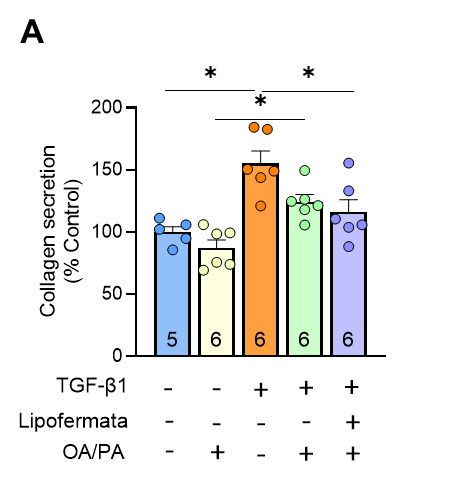


**Supplementary Figure 2. Effect of oleate palmitate mix on collagen secretion.**

**(A)** Collagen secretion in the absence or presence of oleate: palmitate mix (0.5 mM) and TGF-β1 and in the or absence of presence of Lipofermata. Significance set a P<0.05 and assessed by one-way ANOVA with Holms-Sidak multiple comparisons. N=5-6/group and listed in each column. Data are presented as mean ± SEM.


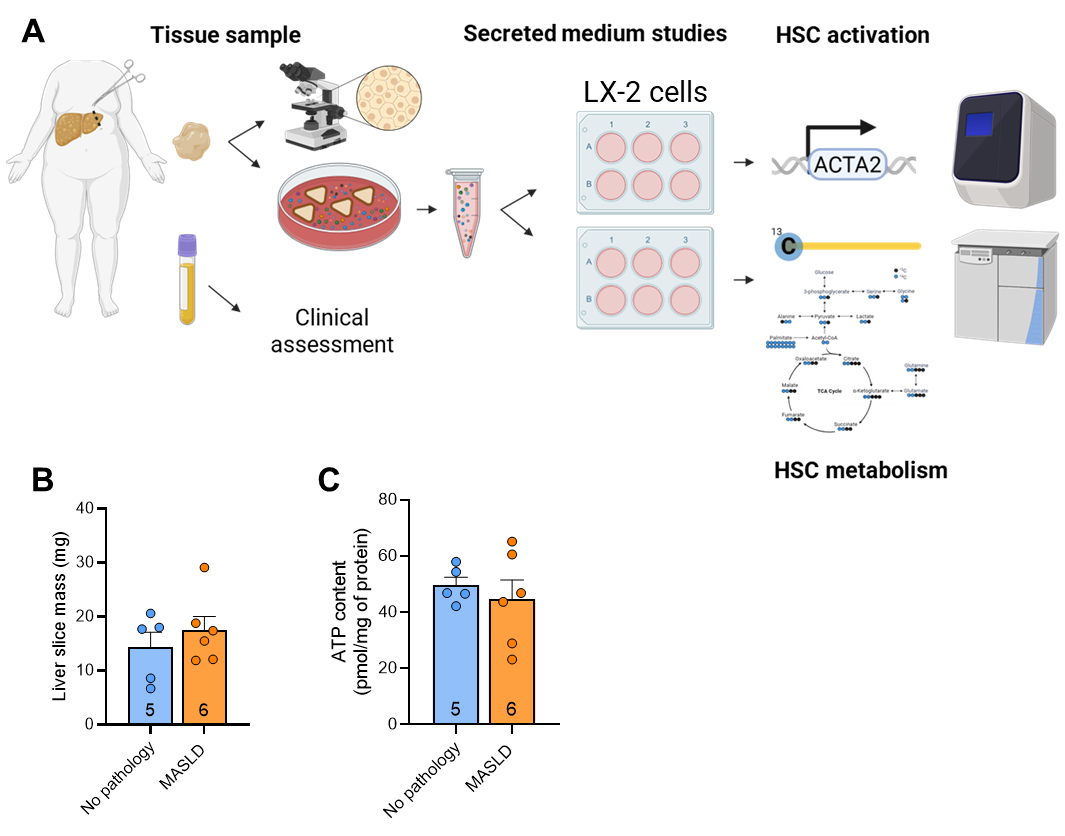


**Supplementary Figure 3. Human liver-secreted medium workflow and precision-cut liver slice viability.**

**(A)** Schematic of study design. **(B)** Mass and, **(C)** ATP content of human liver slices after 16 h incubation period. Significance set a P<0.05 and assessed by assessed by unpaired t-test (Panel B & C) Data are presented as mean ± SEM.


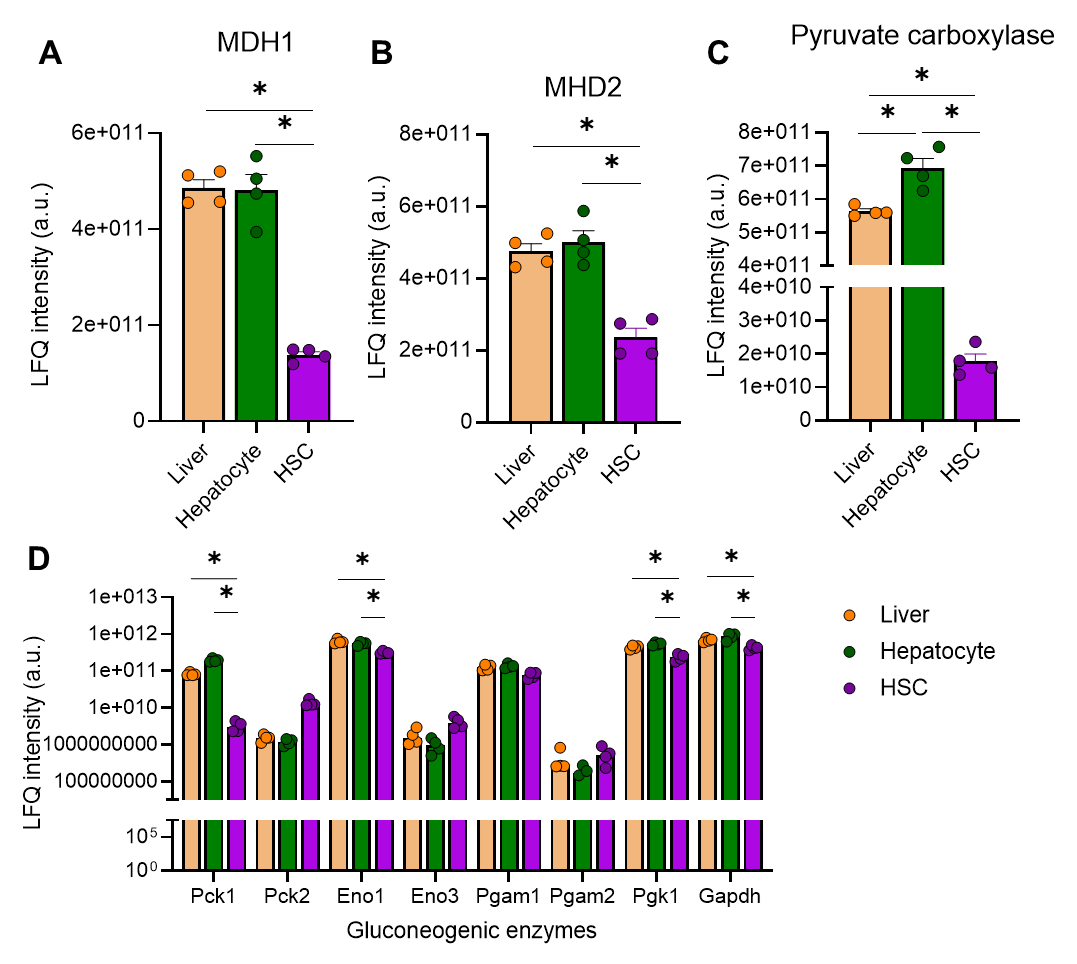


**Supplementary Figure 4. Enzymatic regulators of phosphoenolpyruvate synthesis.**

**(A)** Protein abundance of malate dehydrogenase (MDH1) isoform -1, **(B)** MDH2, **(C)** Pyruvate carboxylase and **(D)** glycogenic enzymes from proteomics of murine liver, isolated hepatocytes and hepatic stellate cells. Data was mined from [36]. Significance set a P<0.05 and assessed by one-way ANOVA with holms-Sidak multiple comparisons. Data are presented as mean ± SEM.
